# Frictions and taxpayer responses: evidence from bunching at personal tax thresholds

**DOI:** 10.1007/s10797-020-09619-0

**Published:** 2020-08-19

**Authors:** Stuart Adam, James Browne, David Phillips, Barra Roantree

**Affiliations:** 1 Economic and Social Research Institute (ESRI) and Trinity College Dublin (TCD), Dublin, Ireland; 2 Institute for Fiscal Studies (IFS), London, England; 3 Tony Blair Institute for Global Change (TBI), London, England

**Keywords:** Behavioural response, Income tax, Social security contributions, Optimisation frictions, Elasticity of taxable income, Bunching, H20, H24, J22

## Abstract

We exploit kinks and notches in the UK personal tax schedule over a 40-year period to investigate how taxpayers respond to income tax and social security contributions. At kinks, where the marginal rate rises, we find bunching by company owner-managers and the self-employed, but not those with only employment income. Responses to notches, where the average rate rises, provide compelling evidence that this is because most employees face substantial frictions: fewer than a quarter bunch even where doing so would increase both consumption and leisure. We develop a new approach for identifying selection in who responds and for decomposing responses into hours and wage components. We find that those employees who do bunch at notches are almost exclusively part-time workers, but tend to have lower wages and work more hours than those part-time workers who do not bunch.

## Introduction

How individuals respond to personal income taxes is of enormous importance for public policy, with both the revenue yield from reforms and the efficiency costs of taxation highly dependent on the magnitude and nature of responses. Since Feldstein (1999) showed that the elasticity of taxable income (ETI) is—under certain conditions—a sufficient statistic for these efficiency costs, a large volume of work has sought to estimate this parameter.

This work typically adopts a panel regression approach (e.g. Auten and Carroll 1999; Gruber and Saez 2002; Kopczuk 2005; Adam et al. 2019). However, such estimates are sensitive to the precise specification used to control for mean reversion or secular trends in income growth, and require a great deal of variation in tax rates over time.^1^ More recently, new ‘bunching’ estimators have been developed which attempt to estimate the ETI by exploiting the prediction of basic neoclassical labour supply models that individuals should bunch at upward kinks and notches in the tax schedule (where, respectively, the marginal and average rates rise at a threshold). While research adopting this design has found evidence of substantial bunching by those (such as the self-employed) with significant scope for income manipulation, tax planning and evasion, evidence of bunching by employees is limited and appears to imply very low elasticities (Kleven 2016).^2^

The primary contribution of this paper is to show that the vast majority of wage earners face substantial frictions to optimising their earnings, sufficiently large to prevent them from bunching at tax thresholds. We interpret ‘optimisation frictions’ broadly, to encompass not only adjustment costs arising from rigidities in contracts or pay structures and search and matching costs, but also inattention, lack of information, optimisation errors, etc. Since such frictions should at least partially abate over time,^3^ it is important to distinguish whether small observed responses reflect frictions or underlying preferences; the unattenuated underlying elasticity will usually (though not always) be the most relevant parameter for long-run policy.

While previous research has suggested that frictions could play an important role in attenuating the responses of employees around tax thresholds, ours is the first to confirm and quantify that effect for a broad group of workers in an advanced economy. Chetty et al. (2011) and Chetty (2012) suggest that frictions could be important but do not actually estimate them. Gelber et al. (2020) estimate the costs of adjusting earnings in the USA but only for low-paid workers approaching retirement, and likewise Zaresani (2019) only for disability insurance recipients in Canada. Kleven and Waseem (2013) look at bunching for a group of relatively high-income workers in the formal sector in Pakistan, finding that around 90% of them do not optimise their earnings in response to a notch due to frictions. Applying this same approach, we show that in an advanced economy, most working-age employees at various points across the earnings distribution face frictions in excess of 2% of earnings, and that for most low-paid workers the frictions are much bigger than that. This is true even for part-time employees, who might be thought to have greater flexibility in their hours of work.

Using high-quality administrative and firm survey data for the UK, we first show that while company owner-managers and the self-employed bunch strongly at kinks in the income tax and social security contributions (SSC) schedule, employees do not. While similar patterns of bunching have previously been identified in the USA (Saez 2010; Mortenson and Whitten 2020), Denmark (Chetty et al. 2011; le Maire and Schjerning 2013), Sweden (Bastani and Selin 2014), the Netherlands (Bosch et al. 2019) and Ireland (Hargaden 2020), the peculiar tax schedule on personal income in the UK, which during the period we study contained notches as well as kinks, allows us to shed light on why.^4^ By creating a strictly dominated region of earnings that no one should choose to locate in, regardless of how much they value consumption relative to leisure, notches provide a means of measuring the extent to which individuals are constrained from reducing their earnings by optimisation frictions (Kleven and Waseem 2013).

We find no bunching below, and no dip above, several notches located at various points across the earnings distribution, meaning that frictions must be sufficiently large to offset the substantial gains from bunching. While we do see some bunching by employees at a notch lower down the earnings distribution, consistent with an underlying taxable earnings elasticity unattenuated by frictions of around 0.10–0.20, most of those who would locate in the dominated region in the absence of the notch still do so in the presence of a notch. This provides compelling evidence that the reason employees do not bunch at kinks is because a large majority face substantial frictions. This is true even immediately above the notch, where the potential tax saving from bunching was at times as high as 9% of earnings for the employee and a further 10.45% for the employer.

A particularly novel feature of this paper is that the firm survey data we use contain information on employees’ hours of work in addition to their earnings. Exploiting this, we find that the bunching observed at a notch low down the earnings distribution is entirely driven by the behaviour of part-time workers, who make up the majority of individuals at this part of the earnings distribution. Bunching is negligible among full-time workers with similar levels of earnings (and correspondingly lower hourly wages), suggesting that they face much greater frictions than part-time workers. We make a methodological contribution to the bunching literature, showing how these responses can be decomposed into hours and hourly wage components, and that selection in who responds can be identified. Applying this approach, we find that those part-time employees who bunch are higher-hours, lower-wage types than those part-time employees who do not bunch.

We also observe interesting variation in responses across taxpayers. Like Kleven and Waseem (2013) and le Maire and Schjerning (2013), we find that company owner-managers and the self-employed are particularly responsive to taxes, reflecting their greater scope for income manipulation, tax planning and evasion. Moreover, in recent years company owner-managers have become much more responsive than the self-employed. Among low-paid employees, we find that those in the retail and hospitality sectors are much more likely to respond than those in the public sector, and—perhaps surprisingly—that there is little difference in the responses of men and women conditional on working part-time. This raises the question of whether well-documented differences in observed behavioural responses between groups (e.g. men and women, employees and the self-employed) are a result of heterogeneity in preferences or in frictions. If increases in the share of women working full-time rather than part-time mean that they face larger frictions to adjusting their hours of work, that might help to explain why Blau and Kahn (2007), among others, find a decline in the elasticity of labour supply for women over time.

The rest of this paper proceeds as follows:  Sect. 2 describes our institutional setting and data. Section 3 provides graphical evidence of bunching at kinks and notches in the UK income tax and SSC schedules and of how it varies between groups. Section 4 uses the bunching estimators of Saez (2010) and Kleven and Waseem (2013) to estimate elasticities from the bunching observed at kinks and notches, respectively. Section 5 outlines our method for decomposing bunching responses and identifying selection. Section 6 concludes.

## Institutions and data

### Institutional setting

The UK has two main personal taxes on income: income tax, paid by individuals on their earned and unearned income, and National Insurance contributions (NICs), paid by employees and employers on earned income only.^5^ Unusually by international standards, most employees in the UK have their exact tax liability deducted from earnings at source through a pay-as-you-earn (PAYE) system and do not have to submit a tax return.^6^ In fiscal year 2015–2016, both income tax and NICs had piecewise linear schedules, applying above tax-free allowances at standard rates up to a common upper threshold of £42,380 per year and at different rates above that. However, their design has not always been so simple, and their structures over the years provide multiple kinks and notches that we exploit to investigate the responsiveness of taxpayers.

#### Income tax

From the start of the 1990s, the UK operated a relatively simple, annual system of income tax, applied at a starting, basic and higher rate to individual income above a tax-free personal allowance.^7^ Table 1 shows these rates for the period of our analysis (1995–1996 to 2015–2016), along with the personal allowance and the thresholds above which the basic and higher rates applied. Different rates of income tax applied to savings and dividend income, as described in the note to Table 1.

**Table 1:** Income tax rates and thresholds for earned income. *Source*: Tolley’s Income Tax, various years

	Rates (%)				Thresholds (£p.a.)^c^		
Fiscal	Starting	Basic	Higher	Additional^a^	Personal	Basic	Higher
Year					Allowance^b^	Rate	Rate
1995–1996	20	25	40	–	3525	6725	27,825
1996–1997	20	24	40	–	3765	7665	29,265
1997–1998	20	23	40	–	4045	8145	30,145
1998–1999	20	23	40	–	4195	8495	31,295
1999–2000	10	23	40	–	4335	5835	32,335
2000–2001	10	22	40	–	4385	5905	32,785
2001–2002	10	22	40	–	4535	6415	33,935
2002–2003	10	22	40	–	4615	6535	34,515
2003–2004	10	22	40	–	4615	6575	35,115
2004–2005	10	22	40	–	4745	6765	36,145
2005–2006	10	22	40	–	4895	6985	37,295
2006–2007	10	22	40	–	5035	7185	38,335
2007–2008	10	22	40	–	5225	7455	39,825
2008–2009	–	20	40	–	6035	–	40,835
2009–2010	–	20	40	–	6475	–	43,875
2010–2011	–	20	40	50	6475	–	43,875
2011–2012	–	20	40	50	7475	–	42,475
2012–2013	–	20	40	50	8105	–	42,475
2013–2014	–	20	40	45	9440	–	41,450

Note: Different tax rates apply to income from savings and dividends. The basic rate of tax on savings income has been 20% since 1996–1997, while the 10% starting rate continued to apply to some savings income until April 2015. After allowing for dividend tax credits, dividends have in effect been taxed at zero (basic rate) and 25% (higher rate) since 1993–1994, with an additional rate of 36.11% from 2010–2011 to 2012–2013 and 30.56% in 2013–2014. When calculating which tax band different income sources fall into, dividend income is treated as the top slice of income, followed by savings income, followed by other income

^a^ Applies to income above £150,000 from 2010–2011 onwards

^b^ From 2010–2011 onwards, personal allowance reduced by 50p for each £1 of income above £100,000

^c^ Lower threshold of total income at which rates shown become payable for those with the standard personal allowance

In 2008–2009, the starting rate was abolished (except for savings income), leaving taxpayers facing either the basic rate (above the personal allowance) or the higher rate (above the higher-rate threshold) on their non-savings, non-dividend income. Subsequent reforms have complicated this rate structure for the c.2% of adults with income above £100,000: since 2010–2011, the personal allowance has been reduced by £1 for each £2 of income above this point, creating a band in which income tax liabilities rise by 60 pence for each additional pound of income (an effective 60% rate) until the allowance is exhausted and the rate falls back to 40%, while incomes above £150,000 have been subject to an additional rate (initially 50%, now 45%).

In summary, the UK income tax schedule contains a number of upwards kinks at which we would expect to see bunching, namely:

- at the personal allowance, throughout
- at the basic rate threshold, until 2007–2008
- at the higher-rate threshold, throughout
- at £100,000 and £150,000, since 2010–2011

In addition, since 2010–2011 there is a downward kink at around £115,000 (where the personal allowance is fully withdrawn and the marginal rate falls back from 60 to 40%), which should result in a dip in the distribution of taxable income analogous to the bunching expected at upwards kinks.

#### National insurance contributions

**Fig. 1:**
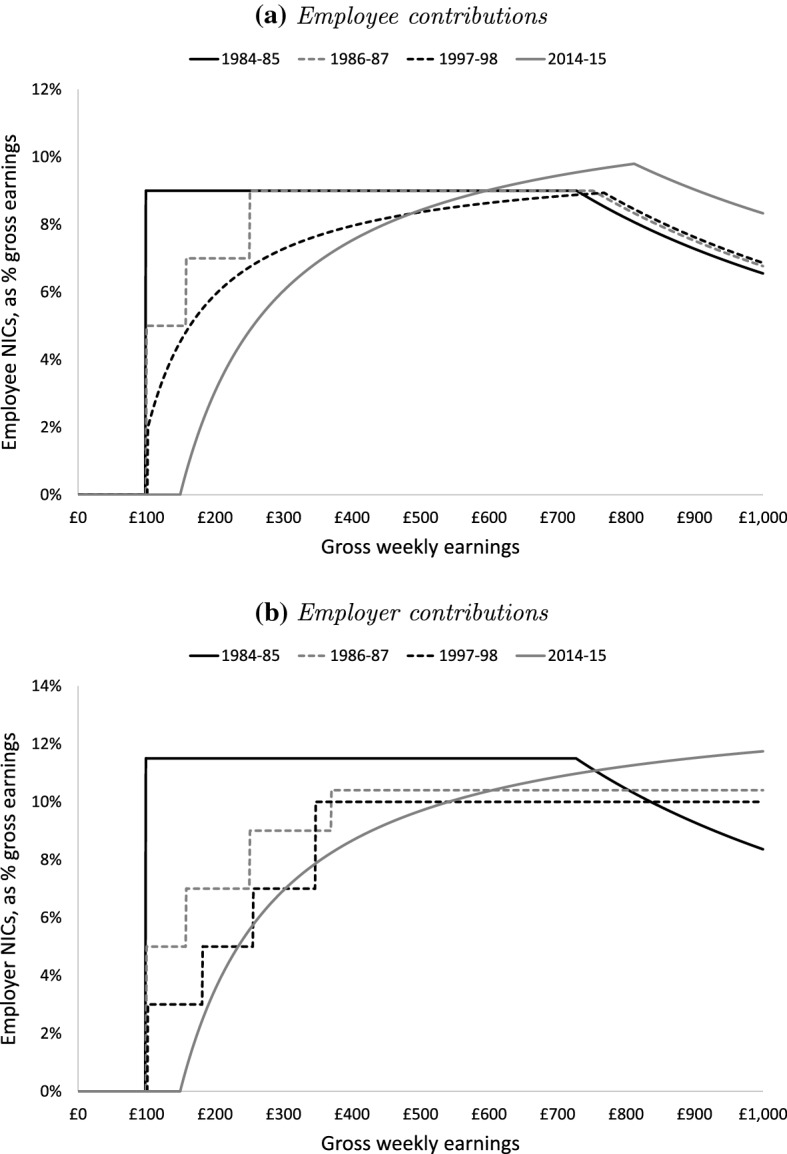
National Insurance contribution schedules (April 2015 prices). *Note* Previous years’ thresholds uprated to April 2015 prices using the retail prices index (RPI). Assumes employee contracted into State Earnings-Related Pension Scheme (SERPS) or State Second Pension (S2P). The 1984–1985 schedule excludes the 1% National Insurance surcharge abolished in September 1984. *Source*: Tolley’s National Insurance Contributions, various years

Between April 1975 and October 1985, once earnings exceeded a lower threshold called the Lower Earnings Limit (LEL), NICs were levied on the entirety of earnings at an employee and an employer rate up to a ceiling called the Upper Earnings Limit (UEL). This created a jump (notch) in NICs liabilities at the LEL and a strictly dominated range of earnings above. The solid black lines in Fig. 1 illustrate this schedule for both employee (panel A) and employer (panel B) contributions in the 1984–1985 tax year.

Reforms taking effect in October 1985 changed the schedule significantly. As shown by the dashed grey lines in Fig. 1, the notch in the employee and employer NICs schedules at the LEL was reduced in size (from 9% and 10.45%, respectively, to 5% apiece), while new notches above the LEL were introduced in both schedules: two in the employee schedule and three in the employer schedule. In addition, the cap on employer contributions at the UEL was abolished.

Another reform, in October 1989, further reduced the size of the notch at the LEL for both employee and employer contributions (to 2%) and eliminated the additional notches above the LEL in the employee NICs schedule. However, it left in place the additional notches in the employer NICs schedule, as shown by the dashed black lines in Fig. 1b.

The structure of NICs in place at the end of our period (shown by the solid grey line in Fig. 1) was arrived at through reforms that took effect between 1998 and 2003. This removed the remaining notches from both the employer and employee NICs schedules, replacing them with kinks at new Primary and Secondary Thresholds for employee and employer contributions, respectively. Employee NICs were also extended for the first time to earnings above the UEL, though at a very low rate (initially 1%, later 2%).

To summarise, the design of NICs creates incentives for taxpayers to bunch:

- below a notch at the LEL from 1975–1976 to 1998–1999
- below multiple notches above the LEL from 1985–1986 to 1998–1999
- at kinks in the employee and employer schedule since 1999–2000

In addition, the zero and reduced rates that have applied above the UEL to employee contributions throughout this period and to employer contributions between 1975–1976 and 1984–1985 create downwards kinks at the UEL which should result in a dip in the distribution of earnings, analogous to the bunching expected at upwards kinks. Tables 6, 7 and 8 in Appendix B contain a full list of these thresholds, along with the size of the notch or kink.

National Insurance was originally envisaged as a ‘true’ social insurance scheme, with a broadly actuarial link between contributions paid and benefit entitlements for each individual. Insofar as there is—or, perhaps, is perceived to be—such a link, National Insurance may not have the same disincentive effects as a simple tax on earnings (Summers 1989). However, in practice the link between contributions and benefits—particularly at the margin—had already been significantly weakened by the 1970s, and had all but disappeared by 2015. For the most part, therefore, NICs acted as a straightforward tax on earnings.

There was one strongly contributory element to the National Insurance scheme. Until very recently, individuals contributing to a private pension could choose whether to ‘contract in’ or ‘contract out’ of the second pillar of the UK state pension system (initially the State Earnings-Related Pension Scheme, SERPS, and later the State Second Pension, S2P). Those contracting out were charged slightly lower rates of employee and employer NICS on earnings between the LEL and the UEL (or, since 2009, the Upper Accruals Point) in exchange for sacrificing future entitlement to SERPS/S2P.

For much of the period, our data do not record whether individuals were contracted in or out. However, the majority of people were contracted out, and the contracted-out rate is arguably a better measure of the ‘tax wedge’ associated with NICs even for those who were contracted in since the rate reduction was a roughly actuarially fair reflection of the forgone entitlements. We therefore use contracted-out NICs rates throughout.^8^ In any case, this does not affect the size of the notch at the LEL, which is crucial for our estimation, since contracting out only reduced the marginal rate between the LEL and the UEL, not the rate charged on earnings below the LEL when the LEL was reached. The marginal rate above the notch plays only a secondary role in our analysis, in translating behavioural responses into elasticities in Sect. 4.^9^

Throughout our period, a much lower rate of employee NICs was available (in exchange for reduced benefit entitlement) to married women who had been claiming it almost continuously since May 1977. Since we cannot identify married women in our data, let alone those eligible for this option and taking it up, we ignore it. The requirement to remain married and in virtually continuous employment since 1977 meant that this affected a large fraction of employed women in the early years of our analysis but very few in later years: the number of women paying it fell from 4.2 million in 1978–1979 to 80,000 in 2000–2001 and 3000 in 2011–2012 (Thurley 2014). We note below where ignoring this reduced rate might significantly affect our results, and check sensitivity to this assumption.

### Data

This paper uses both administrative and employer survey data: the Survey of Personal Incomes (SPI) and the New Earnings Survey Panel Dataset (NESPD).

When looking at income tax thresholds, we use data from the SPI, a stratified random sample drawn from income tax records held by HM Revenue and Customs (HMRC), that cover the tax years between 1995–1996 and 2013–2014.^10^ The sample size increased steadily during that period, from under 60,000 individuals in 1995–1996 to over 700,000 by 2013–2014. Those with very high incomes are oversampled, while the data are not representative of those with incomes too low to pay income tax. For this reason, we do not use the SPI to examine bunching at the income tax personal allowance, from where the starting or basic rate of income tax begins to apply. Income is recorded annually and includes almost all sources that are liable for income tax. One exception is interest and investment income for starting or basic rate taxpayers who do not submit a self-assessment return. This is instead imputed in the SPI, based on administrative data on the aggregate amount and numbers receiving it and on survey evidence on the broad pattern across the income distribution. While the imputation will not allocate the income to individuals accurately-so there is some measurement error in our income variable-the imputed distribution should be a fair approximation, and suggests that savings and investment income amounts to less than £500 for over 90% of employees around the higher-rate threshold (roughly the same fraction as we observe for the self-employed, who must report such income on their tax return). This should not be enough to have a significant impact on our elasticity estimates (which are based on examining bunching in a range of £700 either side of the threshold) unless the employees who in reality bunch happen also to be the few with unusually high savings and investment income, which will not be imputed to them so their bunching will not be visible in our data.

To look at NICs we use the NESPD, a mandatory survey of employers that collects information on employees’ basic characteristics and earnings for a pay period each April, from 1975 to 2015.^11^ The target sample frame is employees whose National Insurance number ends with a specific pair of digits.^12^ In principle, this should deliver a 1% random sample of employees, but in practice it delivers around 0.7% due to non-response and the exclusion of non-civilian employees. At around 165,000 individuals per year, the NESPD contains a far larger sample than UK household surveys and does not suffer from the same degree of measurement error, as responses are provided by employers directly from or with reference to their payroll records.

As well as the length of its coverage, a key advantage of the NESPD is that the earnings measure corresponds closely to the tax base for NICs, most notably recording earnings in a single pay period (typically a week or month) rather than annually.^13^ Unlike the SPI and other administrative datasets typically used to investigate bunching, the NESPD also records hours of work, which we exploit in Sect. 5.

A feature of the NESPD which complicates our analysis is that the pay period for which earnings are observed is close to the turn of the UK’s fiscal year on 6 April, when changes in NICs rates and thresholds usually take effect. For the vast majority of individuals, the earnings we observe will be subject to the NICs schedule of the fiscal year just beginning. But some years’ data contain individuals who face the schedule of the year just ending.^14^ Bunching below the new year’s threshold may appear diffuse if these individuals bunch below the threshold from the fiscal year just ending.

In addition, the NESPD is potentially susceptible to under-sampling of employees earning below the LEL as employers are not obliged to operate PAYE on these jobs, the records of which are used to identify employees falling within the sampling frame of the survey.

There is evidence to suggest that this is not a major issue by the late 1990s:

- First, if there were systematic under-sampling below the LEL, one would expect a corresponding upwards jump in the density at the LEL after 1999, when the NICs exemption threshold was raised above the LEL (removing the tax incentive to stay below the LEL but retaining it as the threshold for the mandatory reporting of earnings). We observe no such discontinuity.
- Second, comparisons of the NESPD with the Family Resources Survey (FRS) and Labour Force Survey (LFS)-which do not suffer from the same potential issue of under-sampling-suggest that by the late 1990s the proportion of employees with earnings below the LEL was only slightly higher in these household surveys than in the NESPD. Moreover, the proportion just above the LEL was also slightly higher in the FRS and LFS than in the NESPD, which cannot be explained by NESPD coverage issues but could be explained by oversampling of lower earners or underreporting of earnings in the FRS and LFS (see Bound et al. (2001) and references therein for a discussion of earnings underreporting and measurement error in household surveys).
- Third, the UK Office for National Statistics (ONS) says in documentation accompanying the NESPD that a 2004 investigation showed `the impact of this under-coverage on [survey] estimates was very small'.^15^
- Fourth, the ONS also reports that there was little change in the NESPD earnings distribution in 2014 when the PAYE sampling frame moved to ‘Real-Time Information’ and larger employers were required to include all of their employees, not just those above the LEL (Office for National Statistics 2017).

There is less evidence available for the earlier part of the period we analyse. If there was no significant under-sampling by the late 1990s, it seems unlikely that it was a major problem in the early/mid-1990s and then suddenly disappeared; but it may be more of an issue in the 1970s and 1980s. One reason under-sampling below the LEL is not a significant problem in recent years is that employers typically include all their employees on their PAYE scheme, even where it is not required; employers were less likely to do that before widespread computerisation, though anecdotal reports suggest that many still did so (it was often simpler to include them, not least because factors such as variable earnings, job changes and interactions with other income sources meant that they might well be obliged to include them at another time anyway). Comparison of the NESPD with the Family Expenditure Survey (FES) suggests that the issue of under-sampling was more significant in the 1980s than in subsequent decades, though more acute for the very lowest earners than for those just below the LEL (consistent with those quite near the LEL being more likely to need including for other reasons). Insofar as this is an issue, we may understate the extent of bunching at the LEL and we note where this may affect our results in the sections that follow.

## Bunching at kinks and notches

Kinks are defined by a change in the marginal rate of tax at a threshold, such as in income tax schedules where income in higher bands is subject to higher rates of tax. Such tax schedules create a convex budget set that the neoclassical model of labour supply predicts should lead to bunching in the distribution of taxable income (Saez 2010). Optimisation errors, arising from an inability to perfectly control pre-tax income, may mean that this bunching appears as a diffuse mass around, rather than a spike at, the kink.^16^

Notches—where the average rather than marginal tax rate increases discontinuously at a threshold—should lead to bunching below, rather than at, the threshold (Kleven and Waseem 2013). This is because the notch creates a jump in tax liabilities, so those who would have located slightly above in the absence of the notch can now obtain a large tax advantage from a small relocation to below the threshold. Indeed, notches often create a dominated region of earnings that no one should locate in, regardless of how much they value consumption relative to leisure. Because both consumption and leisure can be increased by reducing earnings below, the only reason we should observe anyone in the dominated region is because they are subject to optimisation frictions that prevent them from adjusting their earnings.^17^

Such frictions could take many forms. For example, they could reflect lack of understanding of the tax system; rigidities in contracts, pay structures or wage-setting/bargaining processes based on nominal wages; restricted hours choices available within a firm combined with frictions (such as search and matching costs or specific human capital) that make it costly to move jobs; or minimum wages or other institutional features that make reductions in earnings difficult or unattractive (such as mortgage offers or employer pension contributions being specified as percentages of nominal earnings).^18^

**Fig. 2:**
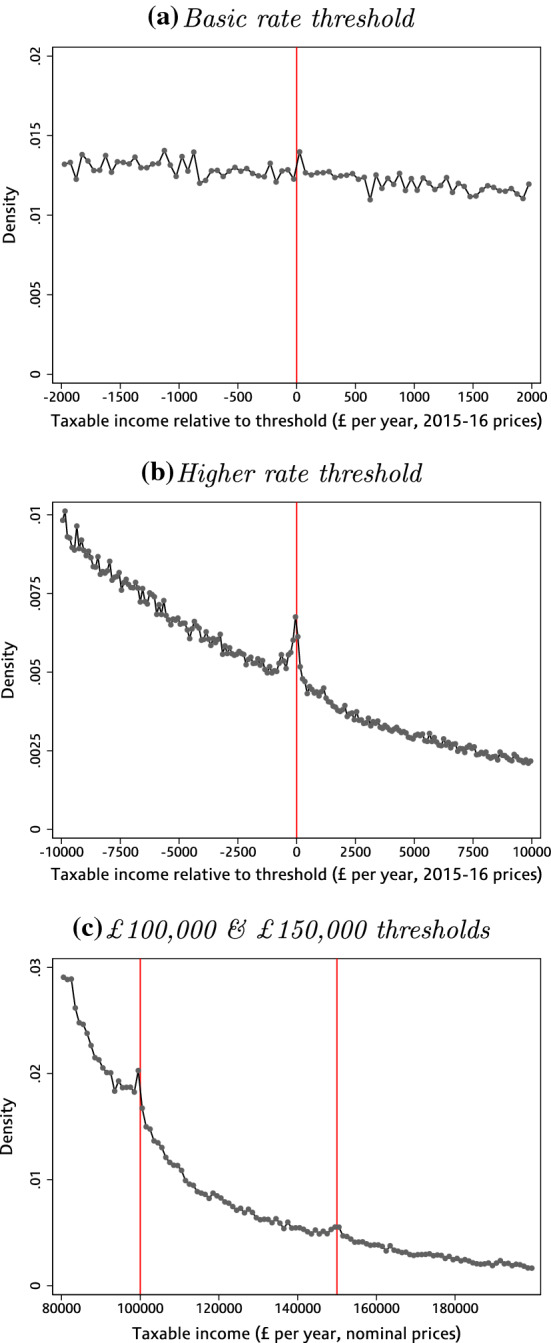
Bunching at upward kinks in the income tax schedule. *Note* Panels a and b show the distribution of annual taxable income in 2015–2016 prices relative to the basic and higher-rate thresholds, respectively, pooling all available years of data between 1995–1996 and 2007–2008 (panel a) or 2013–2014 (panel b). Panel c shows the distribution of taxable income in nominal terms with vertical lines indicating the £100,000 and £150,000 thresholds, pooling the 2010–2011 and 2013–2014 data. *Source*: Authors’ calculations using the Survey of Personal Incomes, 1995–1996 to 2013–2014

In the remainder of this section, we provide graphical evidence of the extent of bunching for different groups at kinks and notches in the UK income tax and NICs schedules. We first look at kinks in the income tax schedule using the SPI data.^19^ Figure 2 shows the distribution of taxable income around the basic rate, higher rate, £100,000 and £150,000 thresholds, pooling observations from all waves of our data. There is little bunching at the relatively small kink created by the basic rate threshold. It is pronounced – if diffuse – at the higher-rate threshold, where the net-of-tax rate (that is, one minus the tax rate) fell by between 20 and 25%. The final panel shows there is clear, but modest, bunching at the £100,000 and £150,000 thresholds.^20^

**Fig. 3:**
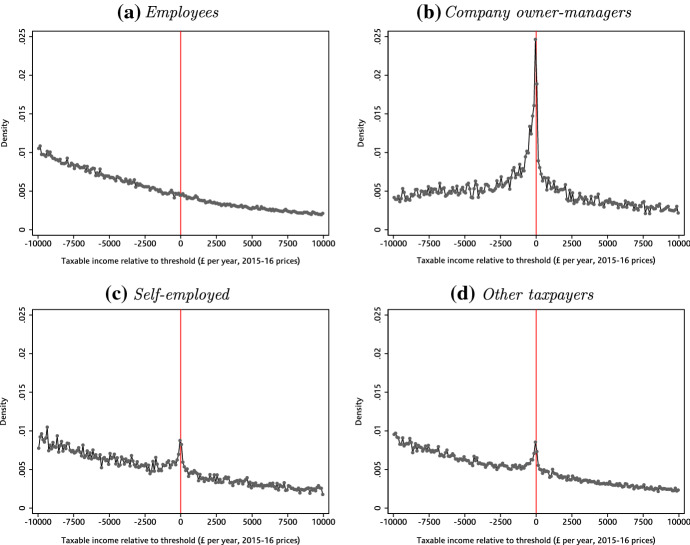
Bunching at the income tax higher-rate threshold, by taxpayer type. *Note* Employees (panel a) are defined as taxpayers whose total income is predominantly (more than 97.5%) derived from employment earnings. Company owner-managers (panel b) are defined as taxpayers who are directors of closely held companies. Self-employed (panel c) are defined as taxpayers whose total income is predominantly (more than 97.5%) derived from self-employment earnings. The other taxpayers group (panel d) contains all remaining taxpayers, and is mostly made up of those with income from a mixture of sources (e.g. earned and unearned income). *Source*: Authors’ calculations using the Survey of Personal Incomes, 1995–1996 to 2013–2014

**Fig. 4:**
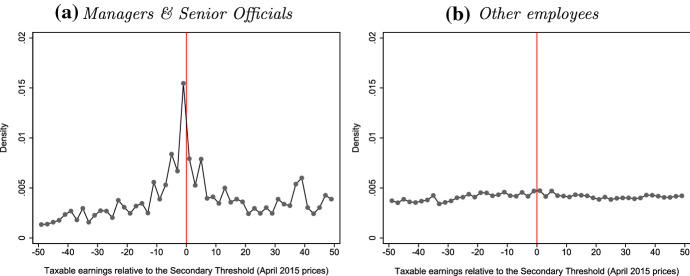
Bunching at the NICs Secondary Threshold. *Note* Groups defined using the Standard Industrial Classification of economic activities (SIC00). Weekly taxable earnings relative to Secondary Threshold. *Source*: Authors’ calculations using the New Earnings Survey Panel Dataset, 2000–2015

What bunching there is at each of these thresholds is mostly due to the behaviour of company owner-managers and (to a lesser extent) the self-employed, with little or no bunching among employees. Figure 3 shows this for the higher-rate threshold, where we saw the strongest bunching; Figs. 9 and 10 in Appendix B show the same results at other upwards income tax kinks. Similarly, Fig. 4 uses data from the NESPD which measures the employment earnings subject to NICs rather than total income for income tax purposes. It shows that we see substantial bunching by managers and senior officials—the standardised occupation category which includes most company owner-managers—at the NICs Secondary Threshold, but nothing for other employees who make up the vast majority of workers at this level of earnings.^21^

There are several possible reasons for the greater responsiveness of those running their own business. They may have more flexibility to adjust their work patterns and, more generally, to fine-tune their income and deductions in any given year, including (for example) by splitting income with a spouse or other family member. They may be more aware of financial planning and more likely to be receiving professional advice. They have more scope to misreport the level or timing of their income and deductions, since they are not subject to the same kind of third-party reporting that employees face on their salary.^22^ And company owner-managers in particular can adjust the amount of profit they retain in the company in order to shift personal income across years (or ultimately take it as capital gains instead). Using newly linked administrative data on personal and corporate tax returns, Miller et al. (2019) show that the last of these possibilities is particularly important in explaining the high responsiveness of company owner-managers documented here.

The lack of bunching among employees might reflect a low underlying behavioural elasticity, or it might reflect frictions that attenuate the response. The patterns of bunching we observe at notches provide robust evidence that frictions play an important role. This is because (as discussed above) notches in the employee NICs schedule create a dominated region that no one should locate in, regardless of how much they value consumption relative to leisure, save for frictions that prevent them from adjusting their earnings. We first examine the notch at the LEL, where between 1975–1976 and 1998–1999 the average rate of employee NICs jumped from 0% to between 2 and 9% of earnings and the average rate of employer NICs jumped from 0% to between 3 and 10.45% of earnings.

**Fig. 5:**
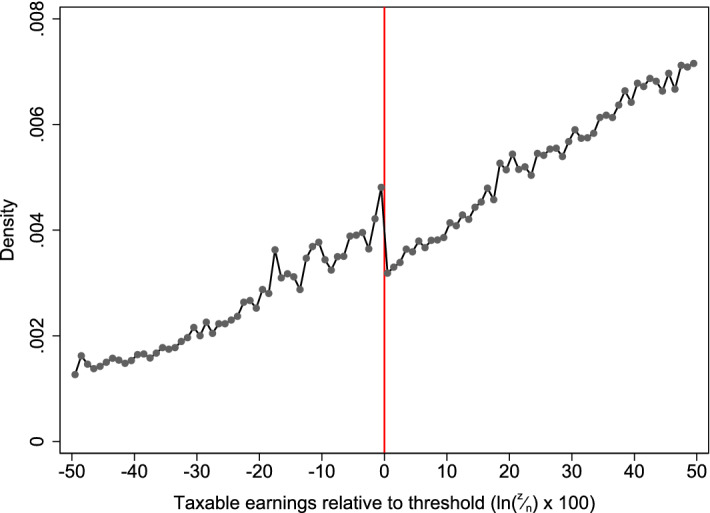
Bunching at the NICs Lower Earnings Limit. *Note* Taxable earnings, *z*, are shown relative to the LEL by plotting the density of observations in bins of $\ln (\frac{z}{\text {LEL}}) \times 100$ so that 0 represents the threshold in each year and 5, for example, means having earnings approximately 5% above the threshold. Excludes individuals with weeklyised or annualised earnings that take common round number values. *Source*: Authors’ calculations using the New Earnings Survey Panel Dataset, 1975–1998

Figure 5 shows the distribution of earnings (*z*) relative to this notch (*n*), pooling all years of NESPD over this period.^23^ There is clear, though modest, bunching below the threshold and a dip above it. But there is also substantial mass visible just above the threshold, in the area that corresponds to the dominated region created by the notch in employee NICs. This provides compelling evidence that the majority of employees at even this low level of earnings are subject to frictions large enough to prevent them from bunching.^24^ As Fig. 11 in Appendix B shows, there is no evidence of any bunching below the notches located higher up the earnings distribution or of any dip above them, even in the dominated region created where the average rates of employee and employer NICs each jumped by 2 percentage points between 1986–1987 and 1989–1990 (panels a and c). This suggests that virtually all employees at these higher levels of earnings faced frictions large enough to prevent bunching.

**Fig. 6:**
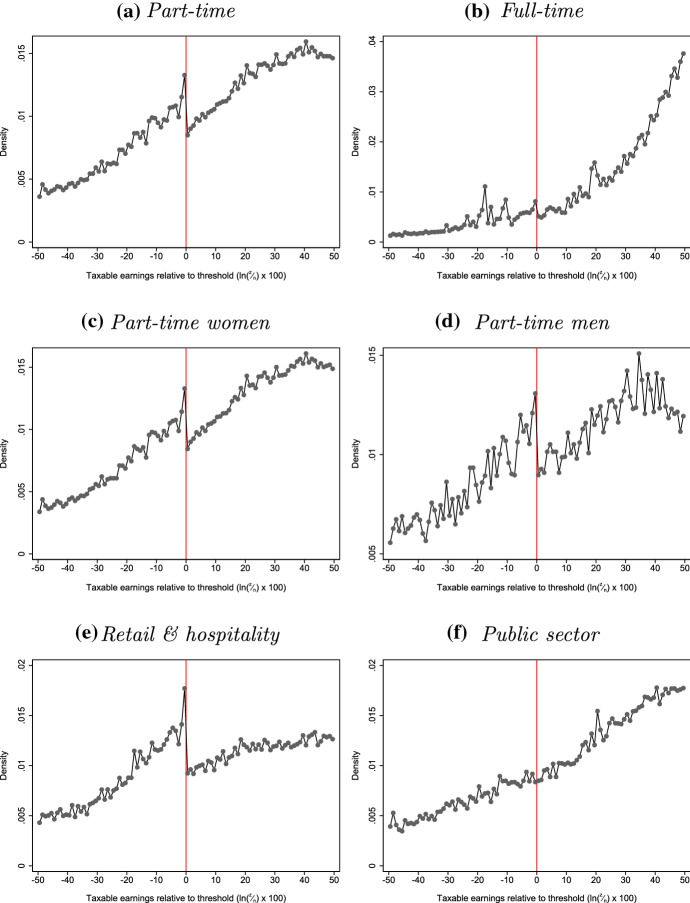
Bunching at the NICs Lower Earnings Limit: by subgroup . *Note* Taxable earnings, *z*, are shown relative to the LEL by plotting the density of observations in bins of $\ln (\frac{z}{\text {LEL}}) \times 100$ so that 0 represents the threshold in each year and 5, for example, means having earnings approximately 5% above the threshold. Excludes individuals with weeklyised or annualised earnings that take common round number values. *Source*: Authors’ calculations using the New Earnings Survey Panel Dataset, 1975–1998

As with kinks, there is substantial heterogeneity in who bunches at the LEL. The first two panels of Fig. 6 show that while part-time employees bunch sharply below the LEL, there is a much less pronounced response among full-time employees. Interestingly, panels c and d show that there appears to be little difference in the bunching responses of women and men conditional upon working part-time (although there are far fewer men working part-time at this level of earnings than there are women). The final two panels show that there are also substantial differences in bunching across industries. Those working in the retail and hospitality sector, where working patterns are typically more flexible (e.g. shift work), bunch sharply below the LEL while there is no observable response among employees in the public sector.

While the presence or absence of bunching at kinks and notches has traditionally been seen as a complication in fitting structural models of labour supply to data (e.g. Burtless and Hausman 1978), recent work has instead viewed it as a potential source of variation that might be used to identify parameters summarising behavioural responses. In the next section, we use the bunching responses documented above to estimate the elasticity of taxable income (or earnings), applying bunching estimators developed by Saez (2010) for kinks and Kleven and Waseem (2013) for notches.

## Estimating elasticities from bunching

Saez (2010) showed that the elasticity of taxable income can be inferred from the income response of the ‘marginal buncher’: the last person who, facing a convex budget set, chooses to locate at, rather than above, a kink *k* where the marginal tax rate rises from $\tau _l$ to $\tau _h$. As this response represents a move between two tangency points, by the definition of the ETI $e \equiv \frac{1-\tau _l}{\tau _h-\tau _l}\frac{\Delta z}{k}$ we have a relationship that depends only on known parameters of the tax schedule ($\tau _l$, $\tau _h$ and *k*) and the income response of the marginal buncher ($\Delta z$). This response can in turn be inferred from the amount of excess mass around the threshold $B \equiv \int _{z_l}^{z_u} (h_0(z)-h_1(z))dz$ (where $z_l$ and $z_u$ define the income range in which bunching is observed and $h_1(z)$ and $h_0(z)$ are the actual and counterfactual distributions of income) as $B = \int _{k}^{k+\Delta z} h_0(z)dz$ (Saez 2010). The crucial step is to estimate the counterfactual distribution of income that would exist in the absence of a kink, $h_0(z)$. We follow Chetty et al. (2013) and estimate $h_0(z)$ by fitting a flexible polynomial to the observed distribution of taxable income, excluding the area [$z_l, z_u$] around *k*.^25^

When the kink is small, this approach identifies the compensated elasticity around the threshold, as the kink does not produce income effects over the bunching segment [$k, k+\Delta z$] (Saez 2010). At larger kinks, without making assumptions about the functional form of utility, the elasticity identified is instead a weighted average of the local compensated and uncompensated elasticities (Kleven 2016). In either case, the bunching estimator easily extends to accommodate heterogeneity in preferences, instead identifying the (compensated, or combined) local *average* elasticity at the threshold.^26^

**Fig. 7:**
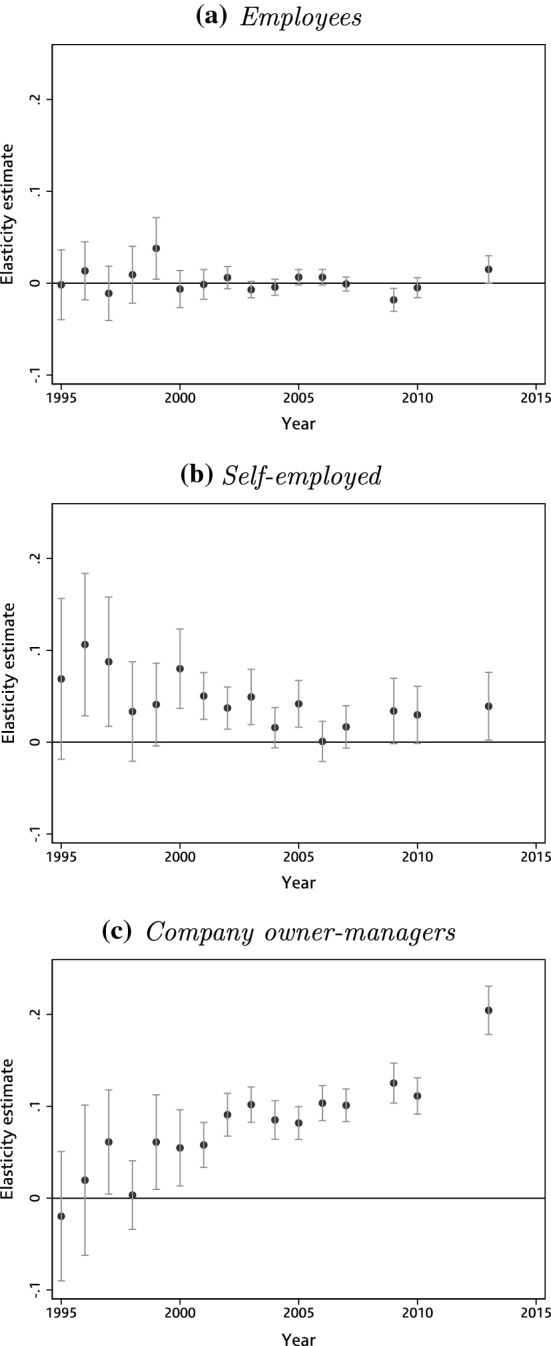
Estimates of the ETI at the income tax higher-rate threshold. *Note* Figures show annual point estimates and 95% confidence intervals for the elasticity of taxable income, obtained by repeating estimation procedure on 500 bootstrap samples, drawn with replacement from the empirical distribution. Employees (panel a) are defined as taxpayers whose total income is predominantly ($\ge 97.5\%$) derived from employment earnings. Self-employed (panel b) are defined as taxpayers whose total income is predominantly ($\ge 97.5\%$) derived from self-employment earnings. Company owner-managers (panel c) are defined as taxpayers who are directors of closely held companies. *Source*: Authors’ calculations using the Survey of Personal Incomes, 1995–1996 to 2013–2014

We apply this estimator to the bunching already documented at the higher rate, £100,000 and £150,000 thresholds, separately for employees, the self-employed and company owner-managers, who each face a different change in tax rates at these thresholds.^27^ Figure 7 shows these estimates at the higher-rate threshold annually for each year covered by our SPI data, along with the bootstrapped 95% confidence interval. The estimates for employees are precisely estimated, and significantly different from zero in only 2 of the 16 years (1999–2000 and 2013–2014). On average, we estimate a higher ETI for company owner-managers (0.078) than the self-employed (0.046), though the coefficient estimates are imprecisely estimated in the 1990s when sample sizes were smaller. As Fig. 7 shows, the relative responsiveness of the two groups seems to have changed over time: our central estimate for the self-employed declines from about 0.10 to almost 0, while that for company owner-managers does the reverse.^28^ The reasons for this apparent gradual decline in the ETI among the self-employed and rise among company owner-managers would be an interesting topic for future research.

Table 2 shows estimates for the same groups at the £100,000 and £150,000 thresholds in 2010–2011 and 2013–2014, the 2 years covered by our SPI data that these thresholds existed. Again, estimates are near zero for employees, and higher for company owner-managers than the self-employed (though both are less precisely estimated than at the higher-rate threshold because sample sizes are smaller).

**Table 2:** Estimate of the ETI at £100,000 & £150,000 income tax thresholds. *Source*: Authors’ calculations using the 2010–2011 and 2013–2014 Survey of Personal Incomes

Year	Employee	Self-employed	Owner-manager
*A. £100,000 threshold*			
2010–2011	− 0.004	0.034	0.093
	(0.004)	(0.019)	(0.016)
2013–2014	0.040	0.045	0.087
	(0.005)	(0.020)	(0.014)
*B. £150,000 threshold*			
2010–2011	0.010	0.100	0.187
	(0.030)	(0.052)	(0.053)
2013–2014	0.059	0.072	0.280
	(0.063)	(0.099)	(0.217)

Note: Bootstrapped standard errors shown in parentheses, obtained by repeating estimation procedure on 500 bootstrap sample, drawn with replacement from the empirical distribution. Employees defined as taxpayers whose total income is predominantly (more than 97.5%) derived from employment earnings. Self-employed defined as taxpayers whose total income is predominantly (more than 97.5%) derived from self-employment earnings. Company owner-managers defined as taxpayers who are a director of a closely held company

Taken together, results from the bunching estimator applied to income tax kinks show clear differences in elasticities, with company owner-managers and the self-employed more responsive than employees. Yet the estimated ETIs are smaller than estimates using different methodologies for high-income individuals in the UK (e.g. Brewer et al. 2010; Browne and Phillips 2017), and toward the lower end of the range estimated in the wider literature (Saez et al. 2012).

However, allowing for even relatively small optimisation frictions could reconcile these estimates with much larger underlying taxable income elasticities. Following Chetty (2012), we illustrate possible effects frictions may be having on our estimates, assuming a quasi-linear utility function and a fixed adjustment cost equal to 1% of utility. Our estimate of 0.204 for company owner-mangers at the higher-rate threshold in 2013–14 is consistent with an elasticity unattenuated by frictions of up to 1.52, while even the smaller estimates for employees and the self-employed at this threshold are consistent with unattenuated elasticities well in excess of one.

If we are interested in obtaining an estimate of the elasticity of taxable income that can be used to predict the response of individuals in a different setting to that in which it was obtained, then it is important to distinguish whether limited bunching (and a correspondingly small estimated elasticity) is the result of low underlying responsiveness or non-trivial frictions. If frictions take the form of fixed costs then we would expect disproportionately stronger responses to bigger tax changes, and if frictions dissipate in the long run then long-run behavioural responses may be larger than short-run responses. And as well as improving our understanding of likely responses to tax changes, frictions may be a policy concern in their own right. Elasticities at least partly reflect utility-maximising behaviour by taxpayers given their preferences between (say) consumption and leisure;^29^ whereas if frictions are *preventing* people from maximising their utility, leading to an inefficient allocation of resources, that might highlight potential gains from policy measures to reduce frictions, ranging from greater information provision to reforms to labour market institutions.

Kinks, unfortunately, provide no means of distinguishing high frictions from low underlying elasticities without variation in the size or location of the kink, and even then only with assumptions on the form frictions take or multiple changes in the size of kinks (Gelber et al. 2020). Notches, however, provide a more promising source of variation for distinguishing these explanations. This is because notches provide an additional empirical moment—the observed density in the strictly dominated region above the notch—that can be used to account for attenuation from frictions.

Kleven and Waseem (2013) show that the ratio of the empirical to the counterfactual density in the dominated region, ${\hat{a}}$, can be used to measure the share of individuals who do not respond to the notch because of frictions. This can then be used to scale up the estimated bunching below the notch (${\hat{b}}$, the difference between empirical and counterfactual distributions in the range $[z_l,n]$) to what it would be if no one were subject to frictions large enough to prevent them from bunching. The authors show that this scaled-up bunching mass, ${\tilde{b}} \equiv \frac{{\hat{b}}}{(1-{\hat{a}})}$, is related to the unattenuated earnings response of the marginal buncher, which can be estimated by calculating the earnings level at which the counterfactual mass between *n* and that point equals the scaled-up bunching mass.^30^

There are then two ways of converting this unattenuated earnings response into a local average unattenuated elasticity. The first is to assume a functional form for utility and use the fact that by definition the marginal buncher is indifferent between locating at the notch *n* and at another higher point, $z^*$. This defines a relationship between known parameters of the tax schedule, the estimated unattenuated earnings response of the marginal buncher $\Delta {\hat{z}}^*$ and the local average (structural) earnings elasticity $e_s$, which can be solved numerically. The second approach is a reduced form one that approximates the effect of a jump in average tax rates as a large change in marginal tax rates. As a notch will induce a larger bunching response than the implicit kink would, this reduced form estimate will be upwardly biased by treating the bunching response as if it were generated by a kink.

As with kinks, the key empirical entity to be estimated is the counterfactual distribution of earnings around the notch. We follow Kleven and Waseem (2013) in estimating this by fitting a flexible polynomial to the number of individuals in small bins of earnings, excluding observations in the range [$z_l,n]$ below the notch that is obviously affected by bunching and an initially arbitrary range [$n,z_{u0}$] above the notch. The polynomial is then repeatedly estimated, increasing the excluded area above the threshold until it reaches a point ($z_u$) where the estimated excess mass between the actual and counterfactual earnings distributions below the threshold, ${\hat{b}}$, equals the estimated missing mass between the actual and counterfactual distributions above the threshold, ${\hat{m}}$. The resulting estimate of the excess mass, ${\hat{b}}$, can then be filled in directly under the counterfactual to estimate the attenuated earnings response of the marginal buncher ($\Delta {\hat{z}}$), or scaled up to account for frictions and then filled in under the counterfactual to estimate the unattenuated earnings response $\Delta {\hat{z}}^*$ and ETI.^31^

We apply this estimator to the bunching observed at the Lower Earnings Limit for three sets of tax years where the jump in average employee NICs rates (and so the dominated region) is of the same size: 1983–84 to 1985–86 (9% points), 1986–1987 to 1989–1990 (5ppts) and 1990–1991 to 1998–1999 (2ppts).^32^ Table 3 shows that both the reduced form (${\hat{e}}_r$) and structural (${\hat{e}}_s$) unattenuated elasticity estimates are greater than zero, but modest. For example, the reduced form estimate is 0.238 for both the 1990–1991 to 1998–1999 and 1986–1987 to 1989–1990 periods, similar to the hours elasticities for low-paid married women over the same time period estimated by Blundell et al. (1998), and to the elasticity of earnings with respect to employee NICs estimated by Adam et al. (2019) using the same data as this paper but exploiting tax changes over time. Both the reduced form and structural estimates for the period 1983–1984 to 1985–1986 are substantially smaller. This may partly reflect the married women’s reduced rate described in Sect. 2.1, which at this time still affected a substantial number of women: if the bunching we observe was generated by a smaller notch than we are assuming, then the true elasticity will be somewhat higher than these estimates.^33^ It may also reflect the potential under-sampling of those below the LEL in the NESPD discussed in Sect. 2.2, though as noted there this is more acute far below rather than just below the LEL.

The elasticities for the period 1990–1991 to 1998–1999 are less precisely estimated, as shown by the large bootstrapped standard errors.^34^ In addition, all of our central estimates are quite sensitive to the exact specification of the fitted polynomial, the range of income over which the counterfactual is estimated, and the excluded range of earnings below the threshold.

The estimated share of individuals who do not respond to the notch because of frictions, ${\hat{a}}$, however, is quite precisely estimated and robust to specification. While these may also be affected by potential under-sampling below the LEL, our estimates suggest that around 75–90% of employees at this low level of earnings are subject to frictions sufficiently large to prevent them from bunching: equivalent to at least 9% of gross earnings in the 1983–84 to 1985–86 period, more than £400 per year in today’s prices. As a result, our estimate of the unattenuated percentage earnings response, $\frac{\Delta {\hat{z}}^*}{n}$, is between 1.5 and 2 times the attenuated one $\frac{\Delta {\hat{z}}}{n}$ obtained solely from the estimated bunching response below the threshold, ${\hat{b}}$.

**Table 3:** Estimates from bunching at the NICs Lower Earnings Limit. *Source*: Authors’ calculations using the New Earnings Survey Panel Dataset, 1983 to 1998

	Central est.	S.E.	*p* value
*Panel A: 1983–84 to 1985–86*			
Reduced form elasticity, ${\hat{e}}_r$	0.085	(0.015)	0.000
Structural elasticity, ${\hat{e}}_s$	0.052	(0.016)	0.002
% attenuated earnings response	11	(1.167)	0.000
% unattenuated earnings response	21	(2.226)	0.000
Excess mass below notch, ${\hat{b}}$	0.090	(0.010)	0.000
Share of individuals facing large frictions, ${\hat{a}}$	0.840	(0.013)	0.000
*Panel B: 1986–87 to 1989–90*			
Reduced form elasticity, ${\hat{e}}_r$	0.238	(0.032)	0.000
Structural elasticity, ${\hat{e}}_s$	0.281	(0.048)	0.000
% attenuated earnings response	10	(0.701)	0.000
% unattenuated earnings response	19	(1.475)	0.000
Excess mass below notch, ${\hat{b}}$	0.153	(0.010)	0.000
Share of individuals facing large frictions, ${\hat{a}}$	0.770	(0.015)	0.000
*Panel C: 1990–91 to 1998–99*			
Reduced form elasticity, ${\hat{e}}_r$	0.238	(0.106)	0.025
Structural elasticity, ${\hat{e}}_s$	0.099	(0.067)	0.140
% attenuated earnings response	12	(4.563)	0.009
% unattenuated earnings response	19	(4.526)	0.000
Excess mass below notch, ${\hat{b}}$	0.086	(0.015)	0.000
Share of individuals facing large frictions, ${\hat{a}}$	0.860	(0.023)	0.000

Note: Bootstrapped standard errors calculated by repeating estimation procedure 1000 times on distribution of earnings drawn with replacement from those shown in Fig. 12. Assumes employees contracted out of the State Earnings-Related Pension Scheme (SERPS) or Second State Pension (S2P) and face NICs schedule shown in Table 6. Excludes individuals with annual or weekly-equivalent earnings that take common round number values

These estimates provide compelling evidence of the important role that frictions can play in attenuating the earnings response of employees, and are reinforced by the complete absence of bunching at notches further up the earnings distribution (Fig. 11, discussed in Sect. 3).^35^ Frictions of this magnitude could also play an important role in reconciling microeconomic and macroeconomic estimates of the compensated elasticity, as suggested by Chetty (2012). Macroeconomic estimates of this parameter are typically derived by calibrating macroeconomic models so that they match cross-country variation in aggregate hours of work, and in general are significantly larger than those obtained with microeconomic data exploiting policy reforms. Chetty suggests that allowing for frictions with utility costs equivalent to 1% of consumption can fully reconcile these differences. Our estimates imply that most low- and middle-earning employees face frictions that are even larger.

## Bunching responses and hours of work

Unlike the administrative data used in most studies of bunching at tax thresholds, the NESPD contains information on hours of work as well as earnings for around 85% of employees sampled. In this section, we show how hours data can be used to reveal more about the nature of bunching responses.

**Fig. 8:**
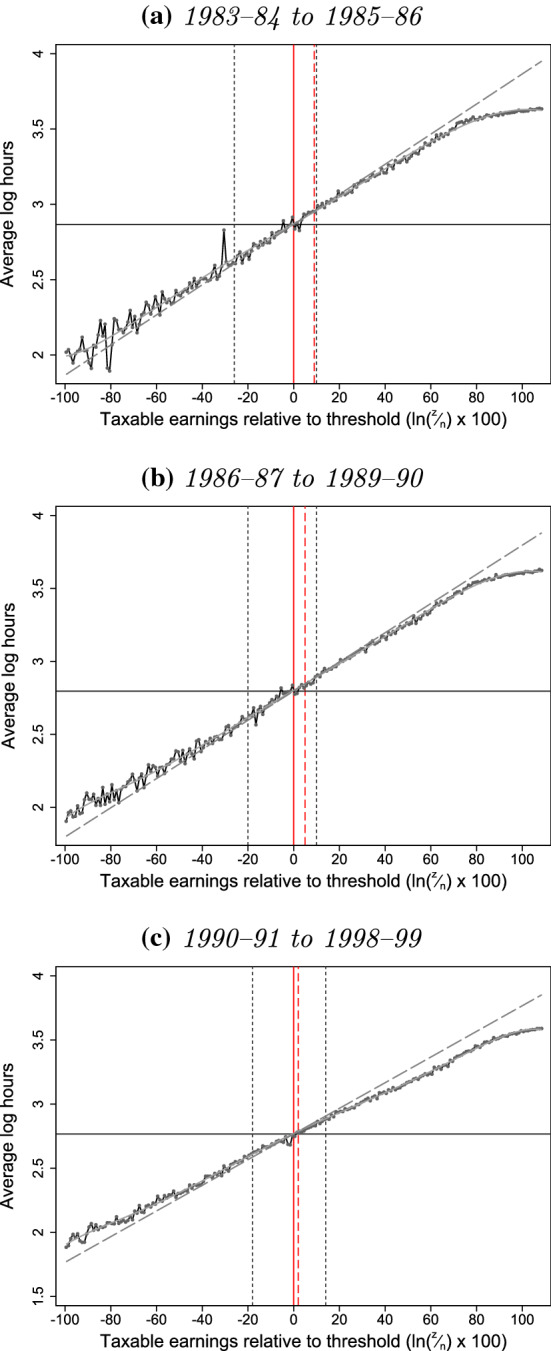
Decomposition of bunching responses at the NICs Lower Earnings Limit. *Note* The horizontal axis shows taxable earnings, *z*, relative to the LEL in terms of $\ln (\frac{z}{\text {LEL}}) \times 100$ so that 0 (shown by the solid vertical line) represents the threshold in each year and 5, for example, means having earnings approximately 5% above the threshold. Overlaid are actual and estimated counterfactual mean log hours. Vertical dotted lines indicate the excluded areas below and above the threshold ($z_l$ and $z_u$, respectively), and the dashed vertical line indicates the top of the dominated region. Excludes individuals with annual or weekly-equivalent earnings that take common round number values, and those missing information for hours. *Source*: Authors’ calculations using the New Earnings Survey Panel Dataset, 1983 to 1998

**Fig. 9:**
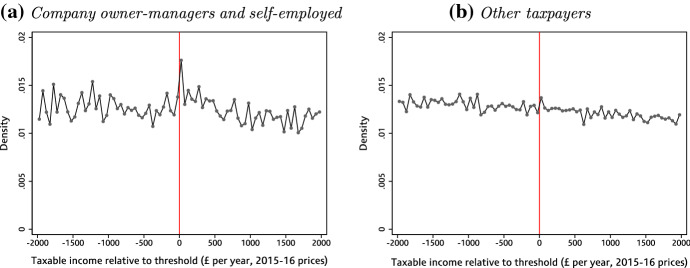
Bunching at the income tax basic-rate threshold. *Note* Company owner-managers defined as taxpayers who are directors of closely held companies. Self-employed defined as taxpayers whose total income is predominantly (more than 97.5%) derived from self-employment earnings. *Source*: Authors’ calculations using data from the Survey of Personal Incomes, 1995–96 to 2007–08

**Fig. 10:**
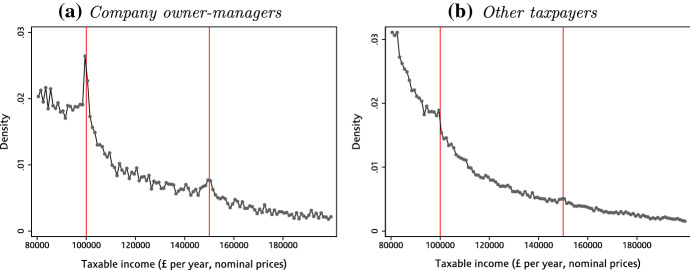
Bunching at the £100,000 & £150,000 income tax thresholds. *Note* Company owner-managers defined as taxpayers who are directors of closely held companies. *Source*: Authors’ calculations using data from the Survey of Personal Incomes, 2010–11 and 2013–14

**Fig. 11:**
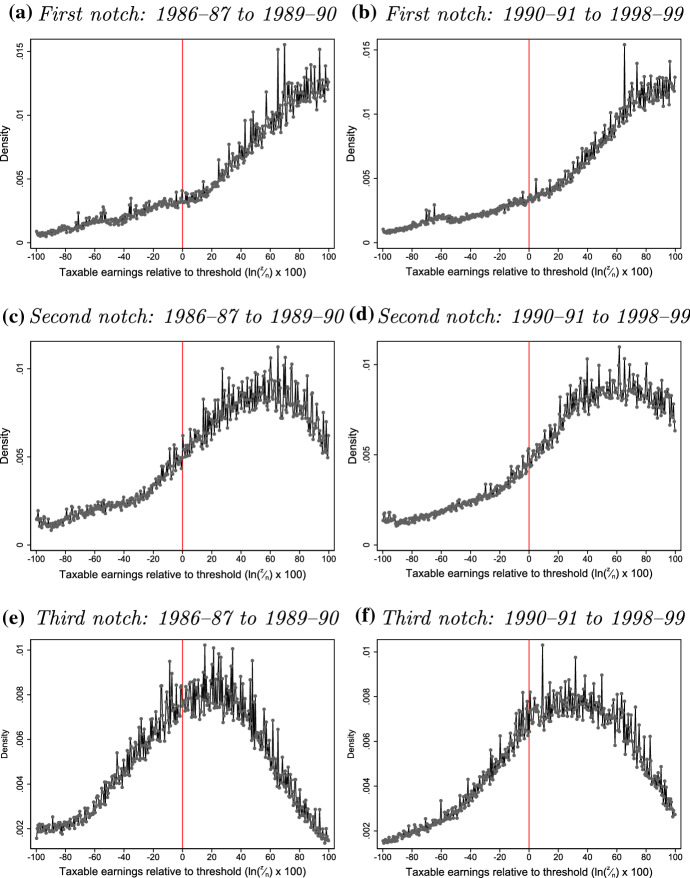
Bunching at NICs notches above the Lower Earnings Limit. *Note* Taxable earnings, *z*, are shown relative to the notch, *n*, by plotting the number of observations in bins of $\ln (z/N) \times 100$ so that 0 (shown by the solid red vertical line) represents the threshold in each year and 5, for example, means having earnings approximately 5% above the threshold. Excludes individuals with weeklyised or annualised earnings that take common round number values, and those missing information on hours. For values of thresholds and sizes of notches see Table 7. *Source*: Authors’ calculations using the New Earnings Survey Panel Dataset, 1986–1998

Figure 8 plots mean log hours in each bin of log earnings around the LEL for the three periods 1983–1985, 1986–1989 and 1990–1998. Since $\ln (z) = \ln (h) + \ln (w)$, this decomposes log earnings at every level of earnings into mean log hours and mean log hourly wage components. We then fit a 7th-order polynomial to the data points excluding those in the interval $[z_l, z_u]$ affected by bunching.

This estimated polynomial allows us to interpolate counterfactual mean log hours in the interval $[z_l, z_u]$ affected by bunching: that is, to estimate what mean log hours (and, by subtraction, mean log hourly wages) at each earnings level would have been in the absence of behavioural responses to the notch. The slope of this counterfactual mean log hours function tells us, before bunching responses, to what extent as we move up the log earnings distribution people had higher earnings because they had higher hours rather than because they had higher wages. Across all three periods, the estimated slope is around 0.9 in the neighbourhood of the LEL: that is, around 90% of the extra (log) earnings of higher earners was because they worked longer hours and only 10% was because of higher (log) hourly wages.^36^ This is precisely estimated and robust to the choice of polynomial order.

Under the assumption that those who do not bunch do not adjust their earnings at all in response to the notch, looking at actual and counterfactual mean log hours at different earnings levels, alongside the actual and estimated counterfactual earnings densities, can potentially tell us about the characteristics and behaviour of bunchers.^37^

### Decomposition of earnings responses

First, we use our estimated counterfactual hours profiles to look at how much of the earnings response of bunchers was through reductions in hours of work rather than hourly wages. The new tax responsiveness literature emphasises that responses to taxation might take the form of reduced hourly wages, rather than reduced hours, because recorded wages capture margins of behaviour such as effort and income shifting. Moreover, the discrete jump in tax liability at notches (unlike at kinks) means that it can make sense to accept lower hourly wages to locate below the threshold even if there is no compensating utility gain in leisure, reduced effort or anything else.

Saez et al. (2012), among others, argue that the responsiveness of taxable income is driven less by hours of work than by factors such as tax evasion, avoidance and income shifting. This conclusion is based mainly on how the taxable income of high-income individuals respond to income tax changes. However, the potential tax-reducing responses to NICs are different from income tax because the tax bases differ, and low-paid workers may respond differently from high-income individuals.^38^

To investigate how employees responded to the notch at the LEL, we estimate how much of the total earnings reduction was accounted for by a reduction in hours. We do this by calculating the reduction in mean log hours as a fraction of the reduction in mean log earnings, using the (observed) actual and (estimated) counterfactual log hours and log earnings across the interval $[z_l, z_u]$ affected by bunching.^39^ Formally, we estimate:

1 $$\begin{aligned} {\hat{h}} \equiv \underbrace{\left( \frac{ \sum _{j=z_l}^{z_u} {\tilde{h}}_{0j} f_{0j} }{\sum _{j=z_l}^{z_u}f_{0j} } - \frac{ \sum _{j=z_l}^{z_u} {\tilde{h}}_{1j} f_{1j} }{\sum _{j=z_l}^{z_u}f_{1j} } \right) }_{\equiv \Delta {\tilde{h}}} \div \underbrace{\left( \frac{ \sum _{j=z_l}^{z_u} {\tilde{z}}_{0j} f_{0j} }{\sum _{j=z_l}^{z_u} f_{0j} } - \frac{ \sum _{j=z_l}^{z_u} {\tilde{z}}_{1j} f_{1j} }{\sum _{j=z_l}^{z_u} f_{1j} } \right) }_{\equiv \Delta {\tilde{z}}} \end{aligned}$$

where ${\tilde{h}}_{1j}$ is the actual and ${\tilde{h}}_{0j}$ the counterfactual mean log hours in log earnings bin *j*; $f_{1j}$ is the actual and $f_{0j}$ the counterfactual number of individuals in log earnings bin *j*; and ${\tilde{z}}_{1j}$ is the actual and ${\tilde{z}}_{0j}$ the counterfactual mean log earnings in log earnings bin *j*.

Table 4 shows that the reduction in mean log earnings is estimated, with reasonable precision, at 0.010, 0.011 and 0.007, respectively, in the three periods 1983–1985, 1986–1989 and 1990–1998. As these are averages across both bunchers and non-bunchers, this implies that those who did bunch reduced their log earnings by 0.244, 0.169 and 0.144, respectively, in these three periods (around 20% on average).^40^

However, the reduction in mean log hours is less precisely estimated, and when combined with that for earnings, results in an estimated share of the total earnings reduction accounted for by a reduction in hours that is associated with very large standard errors. The approach we have developed for decomposing earnings responses into hours and hourly wage responses thus does not appear to have enough power to be informative in our application, though it may yield more interesting results in other cases.

**Table 4:** Estimated share of total earnings reduction through hours. *Source*: Authors’ calculations using the New Earnings Survey Panel Dataset, 1983 to 1998

	Central est.	S.E.	*p* value
*Panel A: 1983–85*			
Change in mean log earnings, $\Delta {\tilde{z}}$	− 0.010	(0.001)	0.000
Change in mean log hours, $\Delta {\tilde{h}}$	-0.010	(0.008)	0.202
% earnings reduction through hours, ${\hat{h}}$	1.088	(0.881)	0.217
*Panel B: 1986–89*			
Change in mean log earnings, $\Delta {\tilde{z}}$	− 0.011	(0.001)	0.000
Change in mean log hours, $\Delta {\tilde{h}}$	− 0.005	(0.006)	0.360
% earnings reduction through hours, ${\hat{h}}$	0.482	(0.530)	0.363
*Panel C: 1990–98*			
Change in mean log earnings, $\Delta {\tilde{z}}$	− 0.007	(0.002)	0.000
Change in mean log hours, $\Delta {\tilde{h}}$	− 0.017	(0.004)	0.000
% earnings reduction through hours, ${\hat{h}}$	2.375	(0.695)	0.001

Note: Bootstrapped standard errors calculated by repeating estimation procedure 1000 times on distributions shown in Fig. 8. Excludes individuals with weeklyised or annualised earnings that take common round number values and those missing information on hours

### Selection of bunchers

Our approach also allows us to identify selection in who responds to the incentives created by the notch at the LEL. To do this, we compare the actual mean log hours of non-bunchers with the estimated counterfactual mean log hours in the region above the notch affected by bunching; that is, we compare ${\tilde{h}}_1$ to ${\tilde{h}}_0$ in the interval $[n, z_u]$. This tells us whether non-bunchers (and, by extension, whether bunchers) were on average high-hours, low-wage workers or low-hours, high-wage workers compared to others with the same counterfactual earnings. Specifically, we test the hypothesis:

2 $$\begin{aligned} \sum _{j=n}^{z_u} f_{0j} {\tilde{h}}_{1j} - \sum _{j=n}^{z_u} f_{0j} {\tilde{h}}_{0j} = 0 \end{aligned}$$

Table 5 shows these estimates of mean log hours, along with the differences between them and the associated standard errors.^41^ These differences are negative throughout, and statistically significant for the periods 1986–1989 and 1990–1998, indicating that those who bunched at the LEL were higher-hours, lower-wage workers than those who did not. We show in Sect. 3 that bunching at the LEL was almost entirely driven by the responses of part-time workers, but this is not inconsistent: since about 90% of workers around the LEL were part-time, the implication of our results is that higher-hours, lower-wage types *among* part-time workers were more likely to bunch than lower-hours, higher-wage types.^42^

Given these estimates, and the counterfactual log earnings densities shown in Fig. 5, we can calculate how much higher bunchers’ mean log hours were than non-bunchers’: 0.129 in 1986–1989, and 0.124 higher in 1990–1998.^43^ To give a sense of magnitudes, simply taking exponentials of these mean log hours suggests that bunchers would typically have worked around 19 h per week in the absence of the notch, whereas non-bunchers with the same earnings typically worked around 17 h per week.^44^

**Table 5:** Actual and counterfactual hours in region above LEL affected by bunching. *Source*: Authors’ calculations using the New Earnings Survey Panel Dataset, 1983 to 1998

	Central est.	S.E.	*p* value
*Panel A: 1983–85*			
Mean log hours of non-bunchers, $\sum _{j=n}^{z_u} f_{0j} {\tilde{h}}_{1j}$	2.911	(0.014)	0.000
Counterfactual mean log hours, $\sum _{j=n}^{z_u} f_{0j} {\tilde{h}}_{0j}$	2.916	(0.010)	0.000
Difference	− 0.005	(0.010)	0.626
*Panel B: 1986–89*			
Mean log hours of non-bunchers, $\sum _{j=n}^{z_u} f_{0j} {\tilde{h}}_{1j}$	2.834	(0.007)	0.000
Counterfactual mean log hours, $\sum _{j=n}^{z_u} f_{0j} {\tilde{h}}_{0j}$	2.849	(0.006)	0.000
Difference	− 0.015	(0.007)	0.025
*Panel C: 1990–98*			
Mean log hours of non-bunchers, $\sum _{j=n}^{z_u} f_{0j} {\tilde{h}}_{1j}$	2.819	(0.036)	0.000
Counterfactual mean log hours, $\sum _{j=n}^{z_u} f_{0j} {\tilde{h}}_{0j}$	2.830	(0.033)	0.000
Difference	− 0.011	(0.005)	0.036

Note: Bootstrapped standard errors calculated by repeating estimation procedure 1000 times on distributions shown in Fig. 8. Excludes individuals with weeklyised or annualised earnings that take common round number values and those missing information on hours

## Conclusion

This paper has investigated behavioural responses to income tax and social security contributions, exploiting cross-sectional variation created by thresholds in UK tax schedules over a 40-year period. At thresholds where the marginal tax rate fell, we found no sign of a dip in the density of the earnings distribution. But there was clear, if modest, bunching at thresholds where the marginal rate rose, especially the higher-rate income tax threshold. We found that company owner-managers and the self-employed were the most responsive to kinks in the income tax schedule, reflecting their greater scope for income manipulation, tax planning and evasion. In recent years company owner-managers have been much more responsive than the self-employed, whereas if anything the opposite was true in the 1990s.

In contrast, employees did not respond at all to kinks in the tax schedule. Notches, where the average rate of tax changes, provide compelling evidence that the reason for this is that most low-paid employees faced substantial optimisation frictions: while there was some bunching by employees below the notch at the National Insurance Lower Earnings Limit in the 1980s and 1990s, only a minority responded in this way. In some years, this meant non-responders paid an additional 9% of total earnings in employee contributions and a further 10.45% in employer contributions. Frictions of this magnitude suggest that long-run responses, or responses to large reforms, could be larger than implied by elasticities estimated from short-term responses to small tax differentials, and could play an important role in reconciling micro- and macro-based estimates of labour supply elasticities, as suggested by Chetty (2012).

We found that there was substantial heterogeneity in which employees bunched at the LEL. Those employed in the hospitality or retail sectors were far more likely to respond than those working in less flexible sectors. Part-time workers were more likely to bunch than full-time workers, though those part-time workers who did bunch were typically higher-hours, lower-wage types than those who did not. Notably, we found little difference in the responses of women and men conditional upon working part-time. This raises the question whether well-documented differences in observed behavioural responses between groups (e.g. men and women, employees and the self-employed) are a result of heterogeneity in underlying preferences or in frictions faced. If increases in the share of women working full- rather than part-time mean that they face larger frictions to adjusting their hours of work, that might help to explain why Blau and Kahn (2007), among others, find a fall in the elasticity of labour supply for women over time. Deepening our understanding of the nature of the frictions we highlight in this paper is not only crucial for integrating disparate evidence on elasticities into a sophisticated understanding of taxpayer responses, but may also have important policy implications in its own right, since to the extent that frictions prevent people from maximising utility, addressing their underlying causes may provide significant welfare gains.

## Figures and Tables

**Fig. 12:**
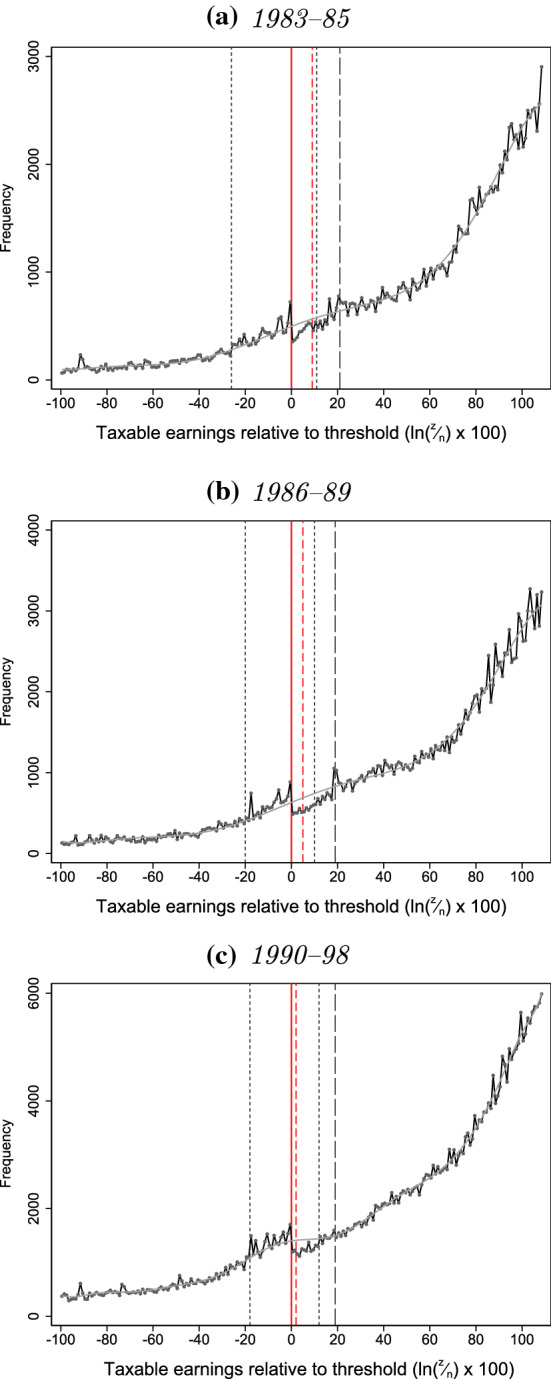
Bunching at the NICs Lower Earnings Limit and estimated earnings responses. *Note* Taxable earnings, *z*, are shown relative to the LEL by plotting the number of observations in bins of $\ln (\frac{z}{\text {LEL}}) \times 100$ so that 0 (shown by the solid red vertical line) represents the threshold in each year and 5, for example, means having earnings approximately 5% above the threshold. Also shown are the estimated counterfactual distribution of income (in grey) and the unattenuated earnings response ($\Delta z^*$, shown by the long-dashed black line), along with the top of the dominated region ($n + d$, shown by the short-dashed black line)and the excluded areas below and above the threshold ($z_l$ and $z_u$, respectively, shown by the dotted lines). Excludes individuals with annual or weekly-equivalent earnings that take common round number values, and those missing information for hours. *Source*: Authors’ calculations using microdata from the New Earnings Survey Panel Dataset

## Data description: the NESPD

The target sample frame of the New Earnings Survey Panel Dataset (NESPD) is civilian employees in Great Britain whose National Insurance (NI) number ends with a specific pair of digits.^45^,^46^ Since the last digits of NI numbers are allocated randomly to all adults and the NESPD sample uses same pair of digits each year, in principle this should deliver a random 1% panel sample of employees.

In practice, the NESPD is not quite a random 1% sample of employees. In fact, it includes around 0.7% of employees on average over the period (1% of employees in Britain would be around 235,000 per year, not the 165,000 we actually observe). The main reason for this is that, despite supposedly being mandatory, the survey suffers from significant non-response. The valid response rate fell from over 75% in the 1980s to around 60% by 2012.^47^ Non-response reduces sample size and therefore the precision of our estimates, though as noted above our sample remains large. Non-random non-response is unlikely to be an issue for our approach, which relies on identifying a bunching at thresholds: to cause a significant problem response rates would need to differ for those just below and just above the threshold, which we have no reason to suspect.^48^

Two other features of the sample frame are particularly relevant when looking at low earners. One is the potential under-sampling of those below the LEL discussed in Sect. 2.2. In addition, since 2005, employees have been removed from the dataset if their earnings were below £10,000 per year (£11,000 since 2009) and either (a) their job title was ‘Director’, (b) they had the same first initial and surname as the employer completing the survey, (c) they ‘fail the automated National Minimum Wage check’ or (d) their earnings were an outlier for their occupation.^49^ This is an attempt to identify and remove company owner-managers who are manipulating their earnings—for example, taking dividends instead to reduce their tax liability—and are therefore perceived to be producing a distorted picture of the earnings distribution (though in practice these criteria may remove some other employees as well). However, for our purposes, such income shifting may be one of the kinds of response to taxation we might like to capture, and this procedure means that we may understate the extent of bunching by managers and senior officials at the Secondary Threshold shown in Fig. 4.

The main earnings variable recorded in the NESPD measures total cash earnings (including pay for overtime, shift premiums, commission, performance-related pay, etc.), excluding benefits in kind and employer pension contributions but without deducting employee pension contributions, relating to a particular pay period (typically a week or month, but in all cases converted to a weekly equivalent by the data provider). This corresponds closely to the tax base for NICs, which is levied on a very similar definition of earnings and is charged separately in each pay period. The only difference we are aware of relates to benefits in kind:

- Some things we might think of as benefits in kind (broadly those that can be exchanged for cash or are equivalent to cash, such as goods or services bought by the employee but paid for by the employer) are treated like cash in tax law and subject to NICs in full. It is difficult to know whether employers are including those things when they provide earnings measure in the NESPD; if they are not then our earnings measure underestimates taxable earnings.
- Other benefits in kind—the principal ones being company cars and fuel and private medical insurance—were not subject to NICs at all until the 1990s. But the NICs base was gradually broadened to bring more benefits in kind within the scope of employer NICs (employer NICs were applied to company cars and fuel from 1991, and to most other benefits in kind from 2000),^50^ though these benefits in kind remain outside the scope of employee NICs. Thus, from 1991 our earnings measure will be a slight underestimate of low-paid workers’ earnings for employer NICs purposes (though not for employee NICs purposes).

Thus, we may slightly underestimate taxable earnings (or, for some benefits in kind in the latter half of our data, underestimate taxable earnings for employer NICs purposes but not for employee NICs purposes). However, the magnitude of any discrepancy is small and unlikely to have a significant impact on our findings: overall we consider the accuracy of earnings reported in our data to be a strength, not a weakness.

Changes in NICs rates and thresholds usually take effect at the start of the fiscal year. The NESPD collects information each year about earnings and hours of work in the particular pay period that includes the ‘survey reference date’, a specific date in April. The precise date varies from year to year, ranging from 4 April to 29 April. Hence the earnings level reported by the employer in the NESPD will refer to the pay period containing the survey reference date, but the applicable NICs rate will generally depend on whether the amount in question is paid before or after 6 April.

Earnings in respect of the pay period containing a particular date in April may be paid before or after 6 April, so we cannot be certain which fiscal year’s NICs schedule applies to the earnings in our data, and so what contribution cap applies. For example, if the employee’s pay period is the calendar month then the employer will record their April earnings in the survey; but if the employee is paid on the first day of each month then those April earnings will be subject to the NICs schedule for the old fiscal year (ending on 5 April), whereas if they are paid on the 15th day or the last day of each month then their April earnings will be subject to the NICs schedule for the new fiscal year (starting on 6 April). Similar ambiguities can arise for employees with other pay periods, depending on the relationship between the survey reference date, the lengths and dates of pay periods, and the point in the pay period at which earnings are actually paid.

For the large majority of observations in our dataset, the earnings we observe will be subject to the NICs schedule of the fiscal year just beginning, but this will not be the case for all observations (particularly in years when the survey reference date is near the start of April) and we cannot identify those for which it is not true. As the NICs thresholds of the fiscal year just beginning were usually above (and never below) those of the fiscal year just ending (due to the routine uprating of tax thresholds in line with inflation), this means that bunching may appear diffuse, with some individuals bunching below the lower threshold.

## Additional figures and tables

See Figs. 9, 10, 11, 12 and Tables 6, 7, 8, 9.

**Table 6:** NICs Lower and Upper Earnings Limits, 1975–1976 to 1998–1999 *Source*: Tolley’s National Insurance Contributions, various years

Year	LEL	Rate at LEL (%)				UEL	Rate above UEL^a^
	(£p.w.)	Employee		Employer		(£p.w.)	y(%)
		Average	Marginal	Average	Marginal		
1975–1976	11.00	5.5	5.5	8.5	8.5	69	0
1976–1977	13.00	5.75	5.75	8.75	8.75	95	0
1977–1978	15.00	5.75	5.75	8.75	8.75	105	0
1978–1979	17.50	6.5	4	10	7.5	120	0
1979–1980	19.50	6.5	4	10	9	135	0
1980–1981	23.00	6.75	4.25	10.2	9.2	165	0
1981–1982	27.00	7.75	5.25	10.2	9.2	200	0
1982–1983	29.50	8.75	6.25	10.2	9.2	220	0
1983–1984	32.50	9	6.85	10.45	7.85	235	0
1984–1985	34.00	9	6.85	10.45	7.35	250	0
1985–1986	35.50	9	6.85	10.45	6.35	265	0
1986–1987	38.00	5	2.85	5	0.9	285	10.45
1987–1988	39.00	5	2.85	5	0.9	295	10.45
1988–1989	41.00	5	3	5	1.2	305	10.45
1989–1990	43.00	5	3	5	1.2	325	10.45
1990–1991	46.00	2	7	5	1.2	350	10.45
1991–1992	52.00	2	7	4.6	0.8	390	10.4
1992–1993	54.00	2	7	4.6	0.8	405	10.4
1993–1994	56.00	2	7.2	4.6	1.6	420	10.4
1994–1995	57.00	2	8.2	3.6	0.6	430	10.2
1995–1996	58.00	2	8.2	3	0	440	10.2
1996–1997	61.00	2	8.2	3	0	455	10.2
1997–1998	62.00	2	8.2	3	0	465	10
1998–1999	64.00	2	8.4	3	0	485	10

Assumes employees contracted out from the State Earnings-Related Pension Scheme (SERPS) or Second State Pension (S2P) into a defined-benefit private pension scheme. Rates shown are those applying in the April at the start of the fiscal year.

^a^ Marginal rate of employer NICs. The marginal rate of employee NICs above the UEL was zero throughout this period.

**Table 7:** Notches above the LEL in the NICs schedule, 1986–1987 to 1998–1999. *Source*: Tolley’s National Insurance Contributions, various years

	Threshold	Rate applied above threshold (%)			
		Employee		Employer	
Year	(£p.w.)	Marginal	Average	Marginal	Average
1986–1987	60	4.8	7	2.9	7
	95	6.8	9	4.9	9
	140	–	–	6.35	10.45
1987–1988	65	4.8	7	2.9	7
	100	6.8	9	4.9	9
	150	–	–	6.35	10.45
1988–1989	70	4.8	7	3.2	7
	105	6.8	9	5.2	9
	155	–	–	6.65	10.45
1989–1990	75	5	7	3.2	7
	115	7	9	5.2	9
	165	–	–	6.65	10.45
1990–1991	80	–	–	3.2	7
	125	–	–	5.2	9
	175	–	–	6.65	10.45
1991–1992	85	–	–	2.8	6.6
	130	–	–	4.8	8.6
	185	–	–	6.6	10.4
1992–1993	90	–	–	2.8	6.6
	135	–	–	4.8	8.6
	190	–	–	6.6	10.4
1993–1994	95	–	–	3.6	6.6
	140	–	–	5.6	8.6
	195	–	–	7.4	10.4
1994–1995	100	–	–	2.6	5.6
	145	–	–	4.6	7.6
	200	–	–	7.2	10.2
1995–1996	105	–	–	2	5
	150	–	–	4	7
	205	–	–	7.2	10.2
1996–1997	110	–	–	2	5
	155	–	–	4	7
	210	–	–	7.2	10.2
1997–1998	110	–	–	2	5
	155	–	–	4	7
	210	–	–	7	10
1998–1999	110	–	–	2	3
	155	–	–	4	5
	210	–	–	7	10

Assumes employees contracted out from the State Earnings-Related Pension Scheme (SERPS) or Second State Pension (S2P) into a defined-benefit private pension scheme. Rates shown are those applying in the April at the start of the fiscal year

**Table 8:** Kinks in the NICs schedule, 1999–2000 to 2015–2016. *Source*: Tolley’s National Insurance Contributions, various years

	Employee NICs			Employer NICs			UEL (£p.w.)
	Primary Threshold	Rate (%)		Secondary Threshold	Rate (%)		
Year	(£p.w.)	Main	Above UEL	(£p.w.)	Main	Above UEL	
1999–2000	66	8.4	0	83	9.2	12.2	500
2000–2001	76	8.4	0	84	9.2	12.2	535
2001–2002	87	8.4	0	87	8.9	11.9	575
2002–2003	89	8.4	0	89	8.3	11.8	585
2003–2004	89	9.4	1	89	9.3	12.8	595
2004–2005	91	9.4	1	91	9.3	12.8	610
2005–2006	94	9.4	1	94	9.3	12.8	630
2006–2007	97	9.4	1	97	9.3	12.8	645
2007–2008	100	9.4	1	100	9.1	12.8	670
2008–2009	105	9.4	1	105	9.1	12.8	770
2009–2010	110	9.4	1	110	9.1	12.8	844
2010–2011	110	9.4	1	110	9.1	12.8	844
2011–2012	139	10.4	2	136	10.1	13.8	817
2012–2013	146	10.6	2	144	10.4	13.8	817
2013–2014	149	10.6	2	148	10.4	13.8	797
2014–2015	153	10.6	2	153	10.4	13.8	805
2015–2016	155	10.6	2	156	10.4	13.8	815

Assumes employees contracted out from the State Earnings-Related Pension Scheme (SERPS) or Second State Pension (S2P) into a defined-benefit private pension scheme. Rates shown are those applying in the April at the start of the fiscal year

**Table 9:** Estimates of the ETI at the income tax higher-rate threshold. *Source*: Authors’ calculations using the Survey of Personal Incomes, 1995–1996 to 2013–2014

Year	Employee	Self-employed	Owner-manager
1995–1996	− 0.002	0.069	− 0.020
	(0.019)	(0.045)	(0.036)
1996–1997	0.013	0.106	0.020
	(0.016)	(0.040)	(0.042)
1997–1998	− 0.011	0.088	0.061
	(0.015)	(0.036)	(0.029)
1998–1999	0.009	0.033	0.003
	(0.016)	(0.028)	(0.019)
1999–2000	0.038	0.041	0.061
	(0.017)	(0.023)	(0.026)
2000–2001	− 0.006	0.080	0.055
	(0.010)	(0.022)	(0.021)
2001–2002	− 0.001	0.050	0.058
	(0.008)	(0.013)	(0.012)
2002–2003	0.006	0.037	0.091
	(0.006)	(0.012)	(0.012)
2003–2004	− 0.007	0.049	0.102
	(0.005)	(0.015)	(0.010)
2004–2005	− 0.004	0.016	0.085
	(0.004)	(0.011)	(0.011)
2005–2006	0.007	0.042	0.082
	(0.004)	(0.013)	(0.009)
2006–2007	0.006	0.001	0.103
	(0.004)	(0.011)	(0.010)
2007–2008	− 0.001	0.017	0.101
	(0.004)	(0.012)	(0.009)
2009–2010	− 0.018	0.034	0.125
	(0.006)	(0.018)	(0.011)
2010–2011	− 0.005	0.030	0.111
	(0.006)	(0.016)	(0.010)
2013–2014	0.015	0.039	0.204
	(0.008)	(0.019)	(0.013)

Standard errors (in parentheses), obtained by repeating estimation procedure on 500 bootstrap samples, drawn with replacement from the empirical distribution. Employees defined as taxpayers whose total income is predominantly (more than 97.5%) derived from employment earnings. Self-employed defined as taxpayers whose total income is predominantly (more than 97.5%) derived from self-employment earnings. Company owner-managers defined as taxpayers who are directors of closely held companies

## Footnotes

1 See Saez et al. (2012) for a critical review of this new tax responsiveness literature. Adam et al. (2019) exploit the panel dimension of the same data used in this paper to estimate earnings elasticities using a best practice version of this approach, benefiting from an unusually long time period with extensive policy variation.

2 For example, while Chetty et al. (2011) observe substantial bunching by the self-employed around income tax thresholds in Denmark, that by wage earners is much smaller and corresponds to an estimate of the ETI that is very close to 0.

3 While the absence of bunching can be persistent (Tazhitdinova 2015), there is evidence to suggest frictions somewhat fade over time (Paetzold 2019; Zaresani 2019; Gelber et al. 2020). Moreover, depending on the nature of frictions, responses to reforms can strengthen over time even while there remains little bunching (e.g. He et al. 2020).

4 Some papers have examined bunching at kinks in the UK in limited contexts. Britton and Gruber (2019) find little bunching by graduates at kinks in the UK’s income-contingent student loan repayment schedule, Miller et al. (2019) examine bunching by company owner-managers at the higher-rate income tax threshold, and Tazhitdinova (2015) documents similar patterns of bunching to us at some kinks in the UK income tax and SSC schedules in the 2000s. We look at both kinks and notches in the UK income tax and SSC schedules over a 40-year period, using data that are more closely aligned with the base each tax is levied on (see Sect. 2).

5 NICs are also paid by the self-employed, at significantly lower rates, but we do not analyse self-employed NICs in this paper.

6 HM Revenue and Customs estimates that for the 2012–2013 tax year, only 10.74 million out of 30 million income taxpayers had to fill in a self-assessment tax return: primarily the self-employed, those with significant unearned income and those with incomes over £100,000.

7 The vast majority of adults are entitled to this personal allowance, although higher allowances have at times existed for lone parents, older taxpayers and married couples. Our analysis takes account of these different allowances, but for convenience we refer throughout to ‘the’ personal allowance.

8 These rates are contained in Appendix Table 6. We use the contracted-out rate for those contributing to a defined-benefit pension: the contracted-out rate for those contributing to a defined-contribution pension (which was less common) varied by age.

9 Earning above the LEL conveys certain other entitlements. But we do not see any bunching above the LEL after 1999, when the LEL stopped affecting contributions but continued to affect entitlement. This suggests that people did not place a high value on (or did not understand) these entitlements and that our estimates of bunching below the LEL in earlier years should not be strongly affected by these weak contributory links.

10 Data for 2008–2009, 2011–2012 and 2012–2013 are currently unavailable.

11 Appendix A provides more information on this dataset and the features that are relevant to our analysis.

12 The use of a fixed pair of digits from 1975 onwards means that the NESPD constitutes a panel. While this paper’s focus is on the cross-sectional variation created by tax thresholds, Adam et al. (2019) estimate the elasticity of taxable earnings using the panel dimension of the NESPD and reforms to NICs.

13 See Appendix A for further details.

14 See Appendix A for further details.

15 See https://ec.europa.eu/eurostat/cache/metadata/EN/earn_ses2010_esqrs_uk.htm.

16 By the same reasoning, a downwards kink should result in a hole in the distribution of income, which may materialise as a less sharp dip if taxpayers are unable to perfectly control their pre-tax income.

17 Not all notches create dominated regions. In our setting, notches in the employee NICs schedule create a dominated region but notches in the employer NICs schedule do not: the structure of employer NICs—specifically, being levied (unlike employee NICs) on a base that excludes the tax itself—means that there is no range in which an increase in gross earnings (and labour cost) is associated with a reduction in consumption. We should still see bunching at such notches, since there is still a discrete tax advantage to be had from an infinitesimal reduction in earnings.

18 Note that locating in a dominated region requires an explanation beyond frictions in adjusting ‘real’ behaviour such as hours of work, since in the dominated region both employer and employee could gain financially from a reduction in gross earnings without any accompanying change in real behaviour. However, this would require co-operation by both employer and employee and might still be prevented by factors such as contractual rigidities or a binding minimum wage; in such cases, and for those above the dominated region, frictions in adjusting real behaviour can still help to explain a lack of behavioural response.

19 The SPI does not allow us to look at the personal allowance kink because it is not representative of individuals with income below this level, where we would expect bunching to occur.

20 We do not observe any hole or dip in the distribution of income at the downwards kink created by the withdrawal of the personal allowance, even among company owner-managers and the self-employed. We are not alone in this: as Kleven (2016) points out, `no research has found evidence of holes around nonconvex kink points'.

21 Again, we do not see any dip in the distribution at the downwards kink created by the UEL.

22 On the importance of third-party reporting, see Kreiner et al. (2016).

23 For the analysis of notches in the NICs schedule we normalise taxable earnings relative to the LEL by plotting the distribution in bins of $\ln (z/n) \times 100$ so that 0 represents the threshold in each year and positive (negative) numbers means having earnings above (below) the threshold. This natural log normalisation makes little difference to the bunching estimates but allows us to decompose (log) earnings responses into additive (log) hours and (log) hourly wage components in Sect. 5. In addition, because employees tend to be paid in round number amounts, particularly multiples of £10 per week or £100 per year, we follow Kleven and Waseem (2013) in dropping all individuals with weeklyised or annualised earnings that take common round number values to avoid conflating bunching in respond to a threshold with a spike in the distribution of earnings at such an amount.

24 In Sect. 4, we quantify the proportion of employees that are subject to frictions sufficient to prevent them from bunching.

25 Our main estimates use a fifth-order polynomial with an excluded range in 2015–2016 prices of £700 around the higher-rate kink, and £7,000 around the £100,000 and £150,000 kinks. Blomquist and Newey (2017) argue that the choice of these parameters constitutes an assumption about the form of preference heterogeneity, to which estimates may be sensitive. We therefore test the sensitivity of our estimates to varying these choices, and note where they are below.

26 Note that, in the presence of heterogeneity, *k* + $\Delta z$ is not the same as $z_u$: *k* + $\Delta z$ is the mean (elasticity) marginal buncher, used to identify the average elasticity, whereas $z_u$ is the highest (elasticity marginal) buncher, the top of the income range affected at all by bunching which we want to exclude when estimating the no-bunching counterfactual income distribution.

27 Since the estimated elasticity depends on the size of the kink giving rise to bunching, we must estimate it separately for each group or period for which the size of the kink was different. We do not estimate ETIs at the NICs secondary threshold because the size of the kink changes over time and our annual sample sizes are too small to apply the estimator to a single year’s data for the only group we observe bunching at this threshold (managers and senior officials).

28 The difference in elasticities between the two groups is not statistically significant at the 95% level in the 1990s, when sample sizes were smaller, but is highly significant from 2002.

29 Though even the ‘unattenuated’ ETIs we estimate below are not structural preference parameters: they are also functions of the tax base and enforcement, and may highlight the extent of avoidance and evasion opportunities.

30 This approach will in fact deliver a downwardly biased estimate of the unattenuated earnings response. This is because the utility gain from bunching declines with distance above the notch and so the share of the population who are constrained from bunching will be larger further above the notch. As our estimate ${\hat{a}}$ is derived from the dominated region immediately above the notch, it will therefore be downwardly biased.

31 Note that, as with kinks, in the presence of heterogeneous elasticities the earnings of the *highest* buncher ($z_u$, obtained by filling out ${\hat{b}}$ between the actual and counterfactual earnings distributions above the notch), which defines the top of the bunching region excluded when estimating the final polynomial, will be different from the earnings of the *mean* marginal buncher ($n + \Delta {\hat{z}}$, obtained by filling out ${\hat{b}}$ between the* x*-axis and the counterfactual earnings distributions above the notch).

32 While NICs are the only tax or benefit withdrawal rate that changes discontinuously at the LEL, an individual’s effective marginal tax rate below the LEL (*t*) depends on household characteristics not observed in the NESPD. To account for this, we use smaller-scale but richer household survey data along with TAXBEN, the Institute for Fiscal Studies’ tax and benefit microsimulation model for the UK, to calculate the average *t* faced by those below the threshold.

33 If we made the alternative (extreme) assumption that all individuals employed in this period were claiming the married women’s reduced rate, the elasticities implied by the bunching we observe would be 0.115 (reduced form estimate) or 0.095 (structural estimate) rather than 0.085 and 0.052, respectively.

34 Standard errors are calculated by drawing (with replacement) bootstrapped distributions of normalised earnings from the empirical one, and repeating the estimation procedure 1000 times.

35 We cannot formally estimate ${\hat{a}}$ in these cases, since without any bunching we cannot identify an excluded region and estimate a counterfactual density. But the absence of any sign of a dip in the distribution in the dominated region above the notch implies that ${\hat{a}}$ is around 1, i.e. virtually everyone in the dominated region is subject to frictions bigger than the size of the notch.

36 The central estimates for the slope of the polynomial at the LEL are 0.88 for 1983–1985, 0.92 for 1986–1989 and 0.86 for 1990–1998. The standard errors are about 0.02 in each case and the confidence intervals all include 0.9.

37 Throughout this section, we restrict our analysis to the 85% of our sample for whom we observe hours of work as well as earnings. References to the actual and counterfactual earnings densities therefore refer to variants of those shown in Fig. 12 which are re-estimated for this restricted sample. In practice, these look very similar to those for the full sample.

38 To the extent that low-paid workers do respond through tax evasion, avoidance and income shifting, our analysis will capture this in the implied hourly log wage.

39 Actual and counterfactual mean log hours in each earnings bin are weighted by the corresponding (actual or counterfactual) density of earnings in each bin.

40 The earnings reduction among bunchers is given by dividing the change in overall mean log earnings by the fraction of the population in $[z_l, z_u]$ that bunch: e.g. for 1986–1989 $-0.010/0.065=0.169$.

41 In this case we weight both actual and counterfactual mean log hours in the different bins by the same, counterfactual, log earnings density, so that we are isolating whether bunchers’ log hours are different from non-bunchers’ in the same bin, not whether bunchers come disproportionately from low earnings bins within the interval.

42 Consistent with this, if we perform the kind of subgroup analysis shown in Sect. 3 separating out those part-time employees who worked under 16 h per week from those working 16 h or more, we see more pronounced bunching among those working 16 h or more.

43 This is obtained by dividing the change in mean log hours for everyone in $[n, z_u]$ by the fraction of the (counterfactual) population in that region who bunch: e.g. for 1986–89 $0.015/0.116=0.129$.

44 The non-linearity of the log function means that these numbers are only illustrative, not averages.
